# Anti-Melanogenic Effects of L-Theanine on B16F10 Cells and Zebrafish

**DOI:** 10.3390/molecules30040956

**Published:** 2025-02-19

**Authors:** Chih-Li Yu, Haiyue Pang, Zhao Run, Guey-Horng Wang

**Affiliations:** Engineering Research Center of Natural Cosmeceuticals College of Fujian Province, Xiamen Medical College, Xiamen 361023, China; e390701@gmail.com (C.-L.Y.); 201500010339@xmmc.edu.cn (H.P.);

**Keywords:** L-theanine, melanogenesis, B16F10, Zebrafish

## Abstract

L-Theanine, a natural amino acid found in green tea (*Camellia sinensis*) leaves, is known for its diverse psychotropic effects. This study aimed to evaluate the inhibitory effect of L-theanine on melanin production and uncover its regulatory mechanism. We evaluated the anti-melanogenic activities of L-theanine in vitro and in vivo. In B16F10 murine melanoma cells induced by α-melanocyte-stimulating hormone, melanin content and intracellular tyrosinase activity were determined, and melanogenesis-related protein expression and signaling pathways were analyzed by Western blotting. Melanin reduction was further assessed using the zebrafish (*Danio rerio*) test. L-Theanine reduced the intracellular tyrosinase activity and melanin content of B16F10 cells. It also attenuated the expression of melanogenesis-related proteins, such as microphthalmia- associated transcription factor, tyrosinase (TYR), TYR-related protein-1, and dopachrome tautomerase. L-Theanine modulated the protein kinase A (PKA), cAMP responder element binding protein (CREB), phosphorylation of/protein kinase B (Akt), glycogen synthase kinase-3β (GSK-3β), and β-catenin. The antimelanogenic activity of L-theanine (<2 mg/mL) was further confirmed using zebrafish larvae. L-Theanine inhibited melanogenesis by downregulating the PKA/CREB and Akt/GSK-3β/β-catenin signaling pathways. In summary, L-theanine shows potential as a skin-whitening compound, warranting further investigation for its possible applications in cosmetic and pharmaceutical products.

## 1. Introduction

Melanogenesis is the process of melanin synthesis and distribution on melanocytes. It is essential for determining the color of skin, hair, and eyes [1]. Melanin protects the skin from ultraviolet ray damage; however, excessive melanin production can form spots and freckles, and even increase the risk of developing skin cancer [2]. Tyrosinase (TYR) is an important enzyme in controlling melanogenesis in melanosomes, so TYR inhibitors are widely used for skin whitening [3]. Many useful and commercial TYR inhibitors are extensively used in cosmetics or medical treatments [4]. However, some of them can cause skin irritation, contact dermatitis or erythema, peeling, and other adverse effects [5]. Therefore, the discovery of effective and safe TYR inhibitors is an important but challenging endeavor in developing skin-whitening products [6].

Melanogenesis is regulated by several signal-transduction pathways, including the PKA/CREB, Akt/GSK-3β/β-catenin, and mitogen-activated protein kinase (MAPK) pathways [7,8,9]. These signaling pathways regulate microphthalmia-associated transcription factor (MITF) activity, playing a crucial role in the transcription of melanogenesis-related proteins such as TYR, TYR-related protein-1 (TRP-1), and dopachrome tautomerase (DCT), ultimately influencing melanin production [10].

α-Melanocyte-stimulating hormone (α-MSH) is secreted by melanocytes and keratinocytes after UV exposure [11]. When melanocortin 1 receptor combines with α-MSH, it induces adenylyl cyclase (AC) to form intracellular cyclic adenosine monophosphate (cAMP), which in turn activates PKA [7,12]. The phosphorylation of CREB can be activated by PKA at Ser 133 to directly bind to the MITF promoter region and stimulate MITF transcription [12].

The Akt/GSK-3β/β-catenin signaling pathway importantly influences melanogenesis. Akt, activated by PI3K, phosphorylates GSK-3β at Ser9, leading to its inactivation [13]. The phosphorylated GSK-3β enhances MITF binding to the TYR promoter, thereby promoting melanogenesis [14]. β-Catenin, which upregulates MITF expression, is phosphorylated by GSK-3β at Ser33 and Ser37, leading to its degradation via the proteasome [15].

The phosphorylation of the MAPK pathway, including extracellular signal-regulated kinase (ERK), c-Jun NH2-terminal kinase (JNK), and p38, can influence MITF expression [16,17]. Previous studies have shown that inhibiting the phosphorylation of p38 or JNK, or activating ERK phosphorylation, can reduce MITF expression, leading to the downregulation of melanogenesis [18,19,20].

**Figure 1:**
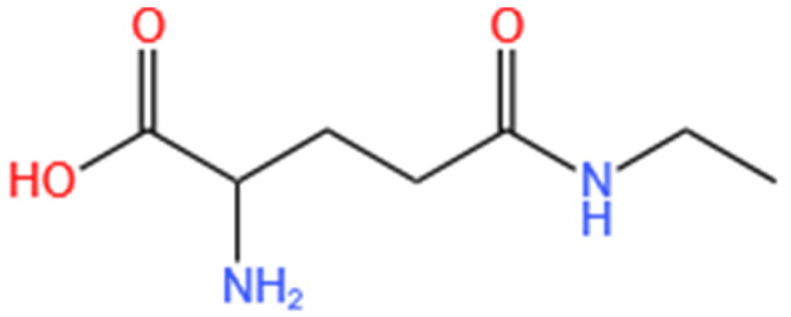
Chemical structures of L-theanine re-drawn using ChemDraw Ultra 11.0 software (Cambridge Soft Corporation, Cambridge, MA, USA).

Theanine (N-ethyl-γ-glutamine) is a non-protein amino acid (Figure 1) found in high concentrations in the leaves of *Camellia sinensis* (green tea) [21]. Recent research has shown that L-theanine exerts various psychotropic effects, including protection against cerebral ischemia–reperfusion injury, stress reduction, anti-tumor activity, anti-aging properties, anti-anxiety, and antioxidant effects [22,23,24]. Despite the anti-melanogenic effects of green tea [25], research focusing on the role of L-theanine in inhibiting melanin production is limited. The present study aims to analyze the melanogenesis-inhibition effect of L-theanine and to reveal its possible regulatory mechanism.

## 2. Results

### 2.1. Effects of L-Theanine on the Viability, Melanin Content, and Intracellular Tyrosinase Activity of B16F10 Cells

**Figure 2:**
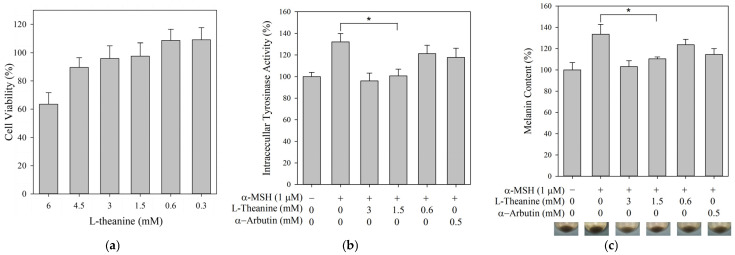
Effects of L-theanine on B16F10 cells. (**a**) Cell viability. (**b**) Intracellular tyrosinase activity. (**c**) Melanin content. The asterisk (*) means *p* < 0.05 compared with the α-MSH group. α-Arbutin served as the positive control.

The B16F10 murine melanoma cell line is a commonly used and well-established model for investigating melanogenesis. The cells can be easily incubated and stimulated with α-MSH to enhance melanin production [8,9,16]. Tyrosinase plays a crucial role in melanin biosynthesis. L-Theanine exerted no inhibitory effect on mushroom tyrosinase activity in vitro. Therefore, B16F10 cells were used to evaluate the effects of L-theanine on melanin content and intracellular tyrosinase activity. To initially assess the potential cytotoxicity of L-theanine, B16F10 cells were incubated with different concentrations of L-theanine (0.3–6 mM) for 24 h. Cell viability was measured using the MTT assay. As shown in Figure 2a, no obvious cytotoxic effects on b16F10 cells were observed at concentrations of up to 3 mM. Therefore, L-theanine concentrations below 3 mM were used for further experiments.

Intracellular tyrosinase activity and melanin content were measured in B16F10 cells for 24 h treatment with or without different concentrations of L-theanine (0.6–3 mM) or α-MSH (1 μM). As shown in Figure 2b,c, L-theanine significantly reduced intracellular tyrosinase activity and melanin content. This suggested that the decrease in melanin content may be due to the reduction in Tyrosinase activity.

### 2.2. Effect of L-Theanine on the Expression Levels of Melanogenesis-Related Proteins in B16F10 Cells

**Figure 3:**
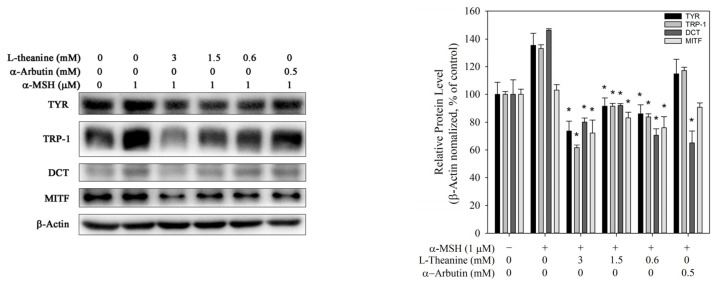
Effect of L-theanine on the expression levels of melanogenic enzymes (TYR, TRP-1, TRP-2, and MITF) in B16F10 cells. α-Arbutin served as the positive control. Western blot analysis showing the protein levels of TYR, TRP-1, DCT, and MITF, normalized against β-actin expression. The asterisk (*) means *p* < 0.05 compared with the α-MSH group.

Melanin production can reportedly be affected by the protein levels of melanogenic enzymes such as TYR, TRP-1, and DCT. MITF, a major transcription factor, regulates the expression of melanogenic enzymes during melanoigenesis [26]. Western blot analysis was performed to investigate the effect of L-theanine on melanogenic enzymes expression in B16F10 cells. The protein levels of TYR, TRP-1, TRP-2, and MITF were significantly decreased by L-theanine (Figure 3). These findings suggested that L-theanine decreased melanin content by reducing the expression of melanogenic enzymes, which was attributed to reduced MITF expression.

### 2.3. L-Theanine Reduced Melanogenesis Through the PKA/CREB Signaling Pathway

**Figure 4:**
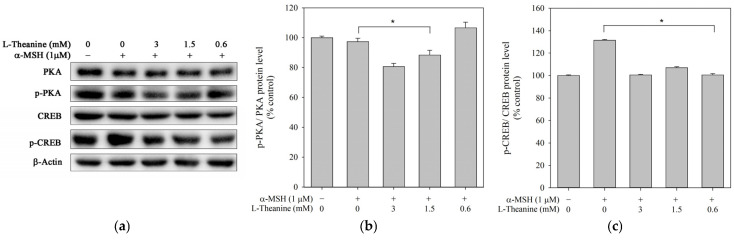
Effect of L-theanine on the protein expression levels of PKA, p-PKA, CREB, and p-CREB in B16F10 cells. Cells were treated with 1 μM α-MSH and different concentrations of L-theanine (0.6, 1.5, and 3.0 mM) at 37 °C for 24 h. The control group was treated without α-MSH and L-theanine. The α-MSH group was treated with 1 μM α-MSH in the absence of L-theanine. (**a**) Representative Western blot images showing the protein expression levels of PKA, p-PKA, CREB, and p-CREB. (**b**) Densitometric analysis of p-PKA/PKA ratio. (**c**) Densitometric analysis of the p-CREB/CREB ratio. Data are expressed as the mean ± standard deviation (SD), based on three independent experiments. The asterisk (*) indicates statistically significant differences (*p* < 0.05) compared to the α-MSH.

α-MSH activated PKA, leading to the phosphorylation and upregulation of CREB in melanocytes. Phosphorylated CREB directly induces the MITF transcription to promote melanogenesis [27]. This study explored whether L-theanine inhibited melanogenesis through the PKA/CREB signaling pathway in B16F10 cells. The results showed that phosphorylated PKA and CREB protein levels were inhibited (Figure 4), suggesting that CREB inhibition was affected by PKA. Thus, the L-theanine-induced downregulation of melanongenesis may be mediated by the PKA-CREB signal pathway.

### 2.4. L-Theanine Reduced Melanogenesis Through the Akt/GSK-3β/β-Catenin Signaling Pathway

**Figure 5:**
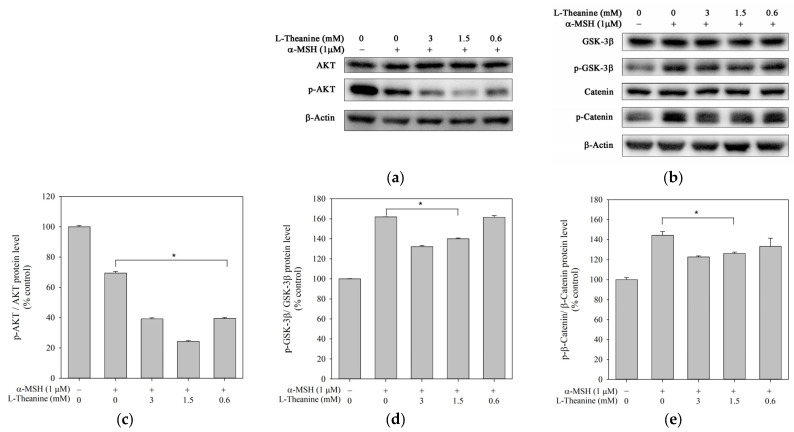
Effect of L-theanine on the protein expression levels of AKT, p-AKT, GSK-3β, p-GSK-3β, β-catenin, and p-β-catenin in B16F10 cells. Cells were treated with 1 μM α-MSH and different concentrations of L-theanine (0.6, 1.5, and 3.0 mM) at 37 °C for 24 h. The control group was treated without α-MSH and L-theanine. The α-MSH group was treated with α-MSH in the absence of L-theanine. (**a**,**b**) Representative Western blot images showing the protein levels of AKT, p-AKT, GSK-3β, p-GSK-3β, β-catenin, and p-β-catenin. (**c**) Densitometric analysis of the p-AKT/AKT ratio. (**d**) Densitometric analysis of the p-GSK-3β/GSK-3β ratio. (**e**) Densitometric analysis of the p-β-catenin/β-catenin ratio. Data are presented as the mean ± SD from three independent experiments. The asterisk (*) indicates statistically significant differences (*p* < 0.05) compared to the α-MSH.

PI3K/AKT and GSK-3β pathways negatively regulate MITF activity, leading to the suppression of melanogenesis [28]. GSK-3β activated by phosphorylation facilitates β-catenin phosphorylation and degradation, leading to a reduction in MITF expression [29]. Accordingly, we examined whether L-theanine inhibited melanogenesis through the Akt/GSK-3β/β-catenin signaling pathway in B16F10 cells. Western analyses revealed that phosphorylated Akt protein level was promoted and phosphorylated GSK-3β and β-catenin protein level was inhibited (Figure 5). The results suggested that the inhibitory effect of L-theanine on melanogenesis may be mediated by the Akt/GSK-3β/β-catenin signal pathways.

### 2.5. Effects of L-Theanine on Melanin Pigmentation In Vivo Zebrafish Assay

**Figure 6:**
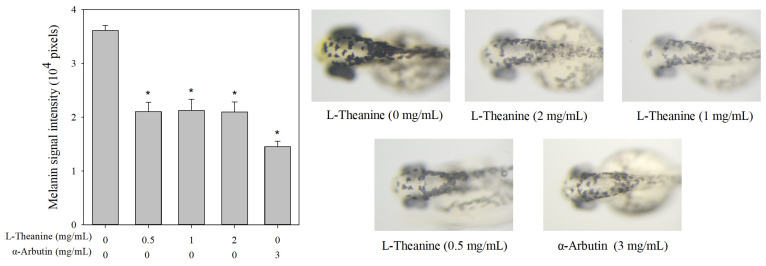
Estimation and comparison of depigmenting effects of L-theanine and α-arbutin by in vivo zebrafish assay. Comparison of depigmenting effects of L-theanine and α-arbutin on zebrafish larvae, illustrating the changes in pigmentation levels. The melanin signal intensity of the control group (0 mg/mL L-theanine) was set. Results are expressed as mean ± SE and represent ten independent tests. The asterisk (*) means *p* < 0.05 compared with the control.

Zebrafish has been established as a key vertebrate model for exploring the depigmentation activities of melanogenic regulatory compounds [30]. To examine the potential effects of L-theanine on melanogenesis in vivo, a zebrafish assay was used. At concentrations below 2 mg/mL, L-theanine did not affect the survival of zebrafish embryos or larvae and has no impact on their development or phenotype (Table S1). Additionally, L-theanine showed a significant inhibitory effect on melanin production in zebrafish compared with the control group, but no significant difference among different concentrations was found (Figure 6).

## 3. Discussion

L-Theanine provides numerous physiological and pharmacological benefits, so it is widely utilized in the food and pharmaceutical industries [31]. Based on the in vitro mushroom tyrosinase activity-inhibition test, L-theanine did not exert any inhibitory effect. B16F10 cells were used to evaluate the inhibitory effects of L-theanine on melanin production. Accordingly, we assessed L-theanine’s potential as a hypopigmenting agent for cosmetic applications.

Melanin levels are directly correlated with either the protein levels or the enzymatic activity of tyrosinase [1]. At concentrations below 3 mM, L-theanine showed no obvious cytotoxic effect on B16F10 cells, whereas melanin content and intracellular tyrosinase activity decreased. L-Theanine also reduced the protein levels of TYR, TRP-1, DCT, and MITF. Several studies have demonstrated that MITF is a key transcription factor to regulate the expression of TYR, TRP-1 and DCT, ultimately controlling melanin production in B16F10 cells [29,32]. These findings suggested that L-theanine inhibited MITF expression, which in turn reduced the levels of TYR, TRP-1, and DCT. The ultimate outcome was decreased melanin production in B16F10 cells.

α-Arbutin is a widely used depigmenting agent in commercial cosmetic products, recognized for its effectiveness in skin whitening and inhibiting melanin synthesis [33]. In this study, the effects of L-theanine on melanin content, intracellular tyrosinase activity and melanogenesis-related protein levels were found to be comparable to those of α-arbutin. This indicates that L-theanine holds promise as a potential alternative to α-arbutin for depigmenting applications.

Previous studies have shown that upstream signaling pathways regulate MITF expression, thereby controlling melanin production in B16F10 cells [18,34]. The upstream signaling pathways involve PKA/CREB, Akt/GSK-3β/β-catenin and MAPKs [35]. L-Theanine further reduced the protein levels of phospholyrated PKA and CREB in α-MSH-induced B16F10 cells. Consistent with Zhou’s finding [36], L-theanine inhibited PKA activity, reduced CREB phosphorylation, and subsequently downregulated MITF expression. Additionally, the Akt/GSK-3β/β-catenin signaling pathway played a role in regulating melanin production [37]. L-theanine increased phosphorylated Akt levels and reduced phosphorylated GSK-3β and β-catenin levels. The effects of L-theanine were similar to previous findings [38,39], showing its ability to modulate the Akt/GSK-3β/β-catenin signaling pathway and thus reduce melanogenesis. In vivo zebrafish assay results reveals that L-theanine can reduce melanin production, similar to Huang’s study [40].

On the other hand, no significant differences were found in the protein levels of phosphorylated ERK, JNK, and p38 MAPKs (Figure S1). The MAPK signaling pathways cannot affect melanin production in B16F10 cells treated with L-theanine.

The above findings suggested that in B16F10 cells, L-theanine regulated melanogenesis-related proteins by modulating the PKA/CREB and Akt/GSK-3β/β-catenin pathways, ultimately reducing melanogenesis. In vivo zebrafish assay results further confirmed that L-theanine can reduce melanin production. Therefore, L-theanine shows potential as a whitening agent in the cosmetic and pharmacy industry. Furthermore, a comprehensive evaluation of the efficacy and safety of L-theanine-induced melanogenesis inhibition is essential, particularly through preclinical studies in animal and human models.

## 4. Materials and Methods

### 4.1. Materials

L-theanine was purchased from Shanghai Macklin Biochemical Co., Ltd. (Shanghai, China). Mushroom tyrosinase, α-arbutin, α-MSH and 3,4-dihydroxyphenylalanine (L-DOPA) were purchased from Sigma-Aldrich (Milwaukee, WI, USA). Primary antibodies against MITF, TYR, DCT, ERK, p-ERK (T202/T185), JNK, p-JNK, p38, p-p38, CREB, p-CREB (S133), GSK-3β, p-GSK-3β (S9), PKC-β, p-PKC-β, β-catenin, and β-actin were purchased from ABclonal (Woburn, MA, USA). TRP-1 was from Abcam (Cambridge, UK). p-β-catenin (S675) was from Cell Signaling Technology (Danvers, MA, USA). All other chemicals, unless specified otherwise, were obtained from Sigma-Aldrich.

### 4.2. Cell Culture

B16F10 murine melanoma cells were purchased from the BeNa Culture Collection (Xinyang, China). The cells were incubated in Dulbecco’s modified Eagle’s medium (DMEM) supplemented with 10% fetal bovine serum and 1% penicillin/streptomycin in a humidified atmosphere with 5% CO_2_ at 37 °C.

### 4.3. Cell Viability Assay

The MTT assay was used to assess the cell viability, with 100 µL of cells (1 × 10^5^ cells/mL) added to 96-well tissue culture plates and treated with various concentrations (0.6–3 mM) of L-theanine for 24 h. After incubation, 10 µL MTT solution (5 mg/mL) was added for 4 h, and the absorbance at 570 nm was measured. The cell viability of the control group (0 µg/mL L-theanine) was set as 100%, and the experimental groups were compared against this control.

### 4.4. Melanin Content and Intracellular Tyrosinase Activity Assay

The cells were treated with 1 μM α-MSH and different concentrations of L-theanine (0.6, 1.5, and 3.0 mM) or α-Arbutin (0.5 mM) at 37 °C for 24 h. The control group received no treatment with either α-MSH or L-theanine. The α-MSH group was treated with α-MSH without the addition of L-theanine. α-Arbutin, a well-known melanogenesis inhibitor, was used as a positive control. After being washed twice with PBS, the cells were lysed in 200 μL of 0.5% Triton X-100 in PBS at 4 °C for 30 min. Following centrifugation at 12,000 rpm for 5 min, the supernatant was removed and stored at −20 °C. The precipitate was solubilized in 200 μL of 1 N NaOH containing 10% dimethyl sulfoxide (DMSO) at 100 °C for 30 min. The absorbance at 475 nm was measured.

The supernatant was used to assess intracellular tyrosinase activity. The supernatant (50 μL) and L-DOPA (5 mM, 150 μL) were mixed in a 96-well microplate. After incubation at 37 °C for 30 min, the absorbance was read at 475 nm.

The supernatant protein was measured by the BCA assay. Melanin content and intracellular tyrosinase activity were calculated and corrected for the concentration of protein. The control was established as 100%, allowing for comparison between the experimental groups.

### 4.5. Western Blotting Assay

The cells were treated as described previously above. Cells were lysed by RIPA buffer and centrifuged at 12,000 rpm for 10 min at 4 °C. The protein (20 μg) was separated with 12% sodium dodecyl sulfate polyacrylamide gel electrophoresis (SDS-PAGE) and transferred to the polyvinylidene difluoride (PVDF) membrane. The PVDF membrane was incubated for 1 h with Tris-buffered saline (TBS) containing 5% bovine serum albumin and 0.1% Tween 20. The membrane was incubated with the primary antibody for 24 h at 4 °C. α-Actin was used as an internal control. After washing twice with TBS containing 0.1% Tween 20, the membrane was incubated with horseradish peroxidase-conjugated anti-mouse or anti-rabbit secondary antibodies for 1 h at room temperature. Protein band detection on the PVDF membrane was performed using NcmECL Ultra reagent (Suzhou, China) and a BIO-RAD ChemiDoc^TM^ XRS+ Imaging System (Hercules, CA, USA). The relative intensity of the protein band was quantified using ImageJ 1.53k software (NIH, Bethesda, MD, USA) and the value was normalized to that of the corresponding loading control. The control was regarded as 100%. * *p* < 0.05 compared to the control group.

### 4.6. Determination of Zebrafish Embryo Mortality Rate

Zebrafish embryos (Hunter Biotech Inc., Hangzhou, China) were collected 24 h post-fertilization and cultured in a constant-temperature incubator at 28 °C, with a 14:10 h light–dark cycle [40]. Culture media containing different concentrations of sample were prepared. A total of 30 zebrafish embryos were tested in each sample. After 72 h of culture, the number of dead embryos was recorded, and their developmental status was assessed. All animal experimental protocols were reviewed and approved by the Medical Ethics Committee of Xiamen Medical College (approval code: 20240306014, Xiamen, China).

### 4.7. Evaluation of Antimelanogenesis Effect in Zebrafish

After assessing the survival rate, it was determined that a concentration of 6 mM L-theanine did not induce death in zebrafish embryos. In total, 30 embryos (6 h post-fertilization) were placed in wells, treated with various final concentrations of the specimens, and incubated at 28 °C for 48 h. α-Arbutin was used as a positive control. Digital images of 10 randomly selected live zebrafish from each experimental group were taken using a stereomicroscope (Olympus SZX7, Hachioji-shi, Japan). The melanin content of the zebrafish larvae was then analyzed using ImageJ software.

### 4.8. Statistical Analysis

The data are presented as the mean ± SEM for in vitro and in vivo experiments. Statistical significance was determined using one-way analysis of variance (ANOVA) followed by Tukey’s test, performed with GraphPad Prism 10 software (GraphPad Software, Inc., San Diego, CA, USA). An asterisk (*) indicates a *p*-value below 0.05, which was considered statistically significant.

## Data Availability

Data are contained within the article or Supplementary Materials.

## Supplementary Materials

The following supporting information can be downloaded at https://www.mdpi.com/article/10.3390/molecules30040956/s1: Figure S1: Effect of L-theanine on the protein expression levels of ERK 1/2, p-ERK 1/2, JNK 1/2/3, p-JNK 1/2/3, p-p38 MAPK and p38 MAPK in B16F10 cells. Cells were treated with 1 μM α-MSH and different concentrations of L-theanine (0.6, 1.5, and 3.0 mM) at 37 °C for 24 h. The control group was treated without α-MSH and L-theanine. The α-MSH group was treated with α-MSH in the absence of L-theanine. (a,b) Western blot showed the protein levels of ERK 1/2, p-ERK 1/2, JNK 1/2/3, p-JNK 1/2/3, p-p38 MAPK and p38 MAPK. Densitometric analysis of (c) p-ERK 1/2/ERK 1/2, (d) p-JNK 1/2/3/JNK 1/2/3 and (e) p-p38 MAPK/p-38 MAPK expressed as the mean ± SD, representing three independent tests. The asterisk (*) means *p* < 0.05 compared with the positive control; Table S1: Number of deaths and mortality rates of *Zebrafish* embryo (n = 30).
