# Supplementary material for: Anti-Melanogenic Effects of L-Theanine on B16F10 Cells and Zebrafish
Source: Molecules. 2025 Feb 19;30(4):956. doi: 10.3390/molecules30040956 (PMC11858779; doi:10.3390/molecules30040956)
Supplement: Supplementary file 1 [file molecules-30-00956-s001.zip › supplymentary data.pdf]

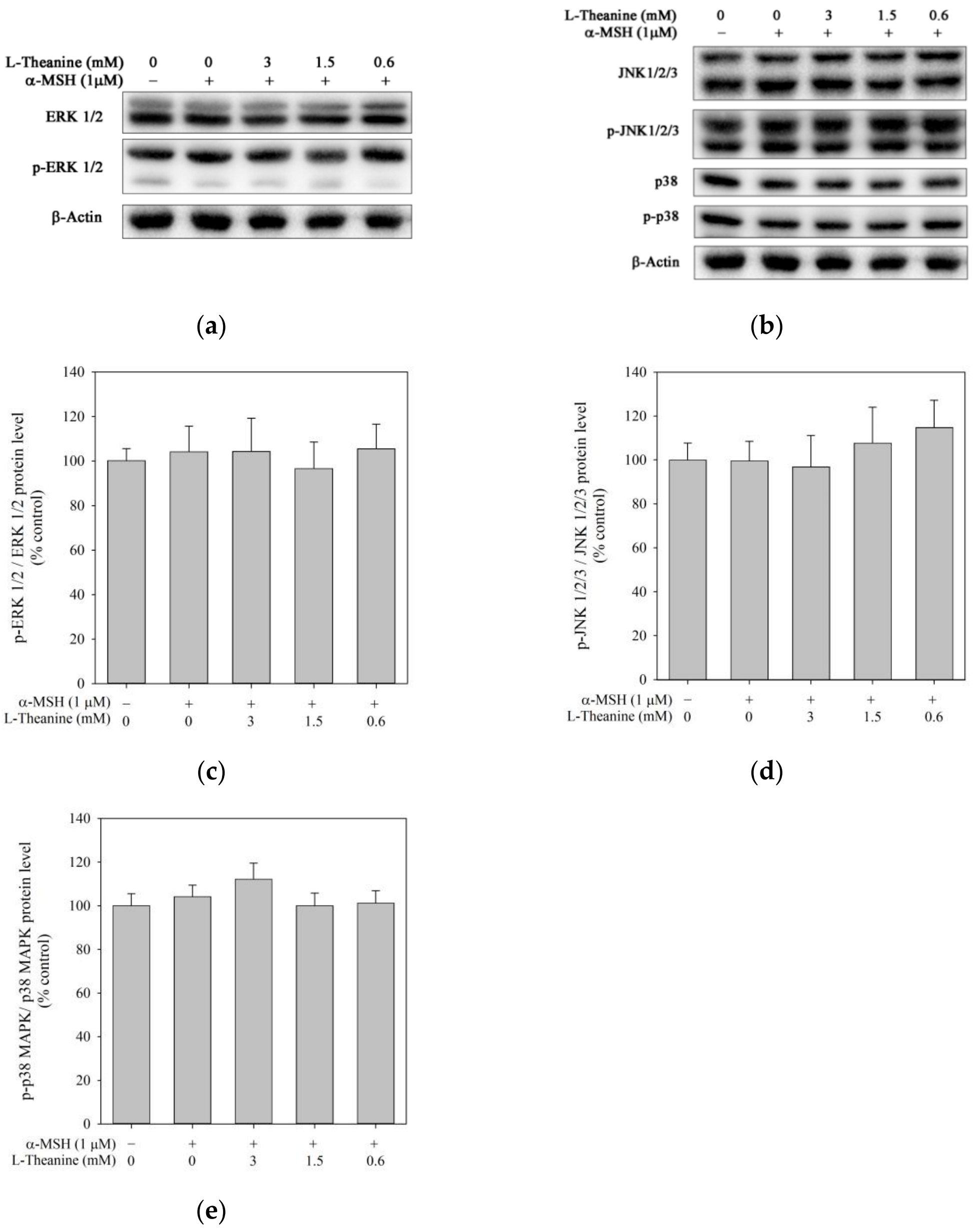

Figure S1: Effect of L-theanine on the protein expression levels of ERK 1/2, p-ERK 1/2, JNK 1/2/3, p-JNK 1/2/3, p-p38 MAPK and p38 MAPK in B16F10 cells. Cells were treated with 1 μM α-MSH and different concentrations of L-theanine (0.6, 1.5, and 3.0 mM) at 37°C for 24 h. The control group was treated without α-MSH and L-theanine. The α-MSH group was treated with α-MSH in the absence of L-theanine. (a, b)Western blot showed the protein levels of ERK 1/2, p-ERK 1/2, JNK 1/2/3, p-JNK 1/2/3, p-p38 MAPK and p38 MAPK. Densitometric analysis of (c) p-ERK 1/2/ERK 1/2, (d) p-JNK 1/2/3/JNK 1/2/3 and (e) p-p38 MAPK/p-38 MAPK expressed as the mean ± SD, representing three independent tests.

Table S1: Number of deaths and mortality rates of Zebrafish embryos (n=30).

Group	Number of deaths (n)	Mortality rate (%)
L-Theanine (0 mg/mL)	0	0
L-Theanine (0.5 mg/mL)	0	0
L-Theanine (1 mg/mL)	0	0
L-Theanine (2 mg/mL)	0	0
α-Arbutin (3 mg/mL)	0	0
